# A case of a long-term survival achieved by surgical treatment and chemotherapy for late recurrence of AFP-producing gastric cancer

**DOI:** 10.1186/s40792-019-0664-z

**Published:** 2019-07-01

**Authors:** Manabu Harada, Hironori Tsujimoto, Takashi Ichikura, Hiromi Nagata, Nozomi Ito, Shinsuke Nomura, Hiroyuki Horiguchi, Yoshihisa Yaguchi, Yoji Kishi, Hideki Ueno

**Affiliations:** Department of Surgery, National Defense Medical College, 3-2 Namiki, Tokorozawa, Saitama, 359-8513 Japan

**Keywords:** Alpha-fetoprotein, Gastric cancer, Recurrence, Long-term survival

## Abstract

**Background:**

Alpha-fetoprotein-producing gastric cancer (AFP-GC) is a relatively rare disease, with a dismal prognosis.

**Case presentation:**

We report the case of a patient with long-term survival after surgery for the recurrence of AFP-GC. A 71-year-old man was diagnosed with gastric cancer and underwent distal gastrectomy with D2 lymphadenectomy (pT3N2M0). Pathological examination of the resected specimen revealed AFP-GC. Fifteen years after the gastrectomy, the patient experienced anorexia and was admitted with a mass located at the mesentery of the small intestine. Following a diagnosis of gastrointestinal stromal tumor of the mesentery, a tumor resection with partial small intestine was performed. The final histopathological diagnosis was AFP-GC’s recurrence in the small-bowel mesentery. Two months later, multiple liver metastases were identified, and serum AFP level was found to be extremely high (17,447 ng/mL). Chemotherapy with S-1+CDDP (SP) was initiated for liver metastasis. However, owing to anorexia and fatigue, SP therapy was discontinued following the patient’s request at the end of two courses. A CT scan at 1 month after the discontinuation of chemotherapy did not reveal liver metastasis, and serum AFP level decreased to the normal range. He is alive at present with no re-recurrence and no elevation of serum AFP level at 7 years after the second surgery without any chemotherapy.

**Conclusion:**

Even if recurrence of AFP-GC is diagnosed, radical resection and chemotherapy are effective, as noted in the present case.

## Background

Alpha-fetoprotein-producing gastric cancer (AFP-GC) is a relatively rare disease, accounting for 1.3–15% cases of gastric cancer [1, 2]. AFP-GC is associated with poor prognosis because of high rates of liver and lymph node metastases [3, 4]. Synchronous or metachronous liver metastases have been frequently observed in AFP-GC, even in the early stage of cancer [1]. Earlier studies have reported that significant prognostic factors in AFP-GC were liver metastasis and pathological stage [5, 6].

Herein, we present a case of a patient with long-term survival after successful treatment with radical surgery for the solitary metastasis of the mesentery and chemotherapy for the multiple liver metastases of AFP-GC at 22 years after the first gastrectomy.

## Case presentation

**Fig. 1:**
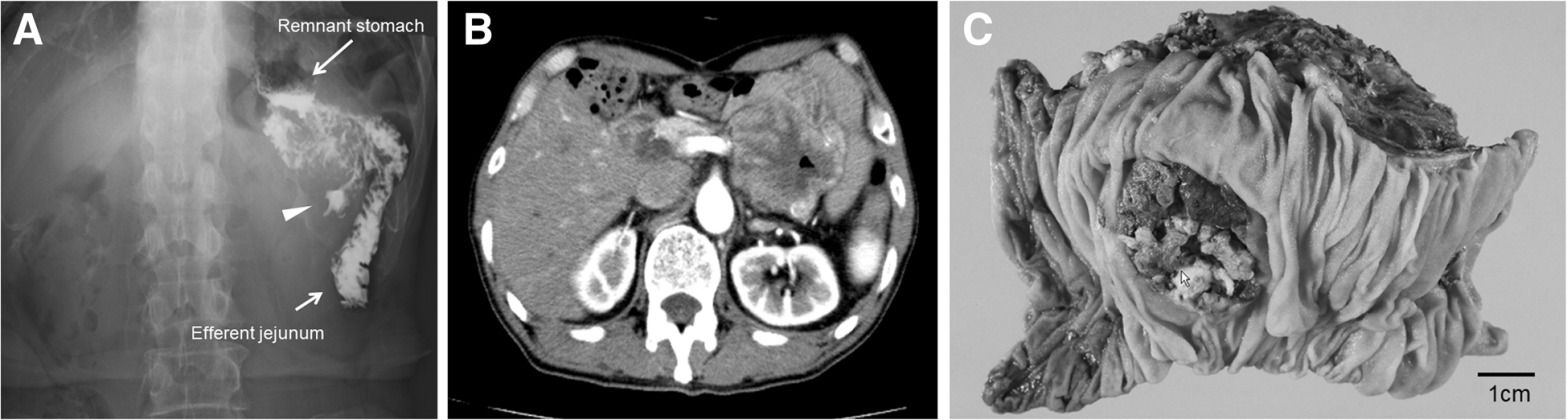
Pictures before surgery. **a** Upper gastrointestinal series revealed the presence of contrast medium in the ulcerative lesion from the afferent jejunum. **b** Abdominal contrast-enhanced computed tomography revealed a 70-mm mass at the mesentery of the jejunum. **c** A 75 × 70-mm-sized mass was found at the mesentery of the jejunum with infiltration of all the layers of jejunum wall in the resected specimen

**Fig. 2:**
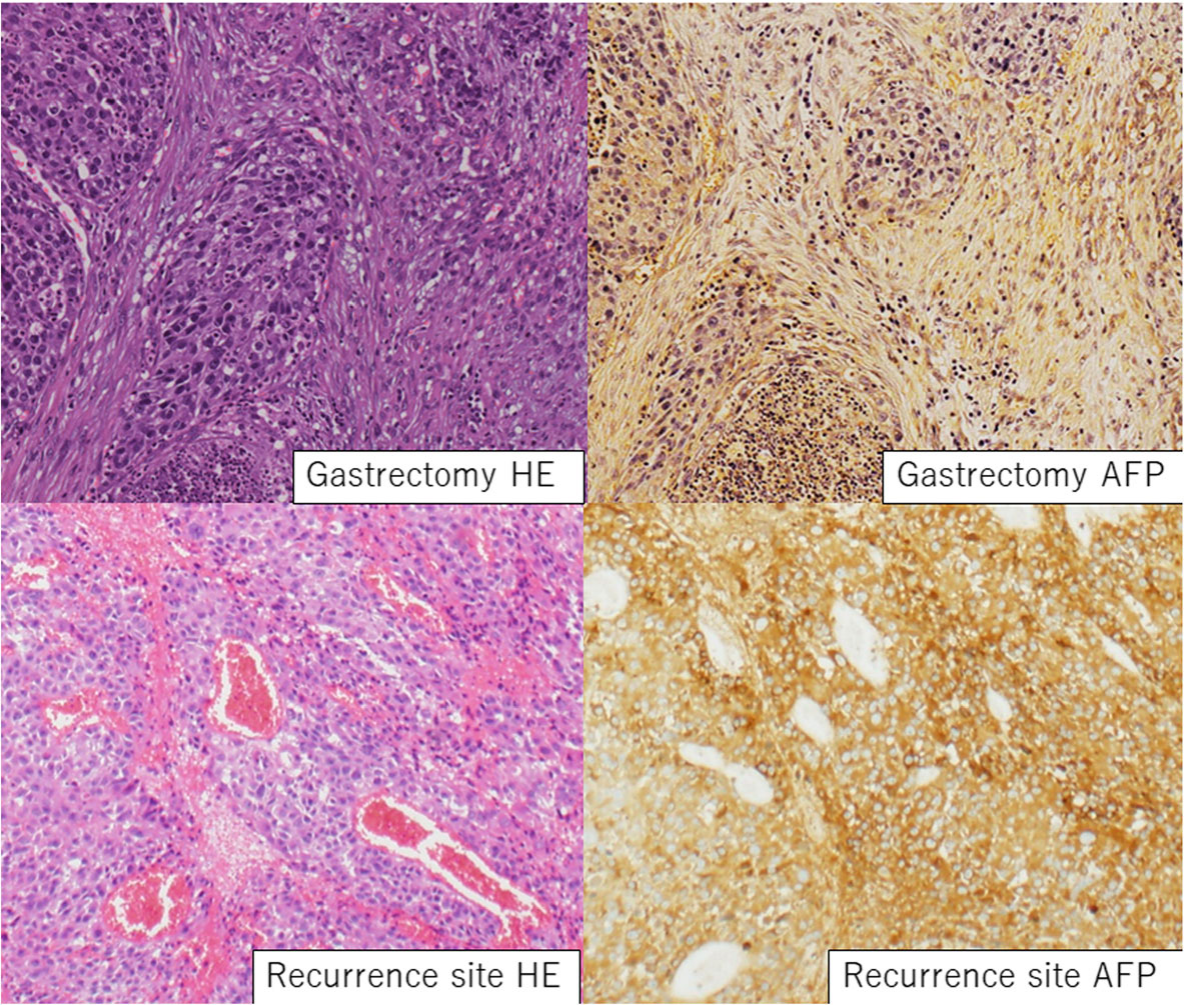
Microscopic findings. Pathological examination indicated that the tumor cells had selling nucleus and acidophilic cells with alveolar growth pattern. Additionally, immunohistochemical examination indicated AFP positivity of the tumor cells, similar to the pathological features of primary gastric cancer resected 15 years ago

A 71-year-old man was admitted to our hospital with complaints of persistent anorexia. Fifteen years ago, he had undergone distal gastrectomy with D2 lymphadenectomy, followed by Billroth-II type reconstruction for gastric cancer. Pathological examination of the gastric tumor revealed an AFP-GC, and the pathological stage was pT3 (SS) N2 M0, stage IIB (Japanese classification of gastric carcinoma: 3rd English edition) [7]. The serum AFP level was 3720 ng/mL (normal range < 10 ng/mL) preoperatively and decreased to 8.0 ng/mL after gastrectomy performed 15 years ago. With the exceptions of the hemoglobin (7.8 g/dL) and serum AFP (17,447 ng/mL) levels, all serum levels tested were within the normal range. Hepatitis B and C were negative, and he did not have any elevated aminotransferases. The levels of carcinoembryonic antigen (normal range < 5.3 ng/mL) and carbohydrate antigen 19-9 (normal range < 37 U/mL) were 4.1 ng/mL and 13.5 U/mL, respectively. Upper gastrointestinal series revealed a contrast medium in the ulcerative lesion from the afferent jejunum (Fig. 1a). Endoscopic examination demonstrated the presence of an ulcerative lesion located at 15 cm from the site of anastomosis of gastrojejunostomy at the afferent jejunum. Pathological examination of biopsy specimens revealed adenocarcinoma consistent with primary gastric cancer resected 15 years ago. An abdominal contrast-enhanced computed tomography (CE-CT) revealed the presence of a 70-mm-sized mass at the mesentery of the jejunum (Fig. 1b). Fluorodeoxyglucose positron emission tomography (FDG-PET) demonstrated the presence of an abnormal accumulation of FDG at the mass without any distant metastasis. Based on these findings, we suspected a metastatic tumor from gastric cancer at the mesentery of the jejunum and performed a laparotomy. Macroscopically, a 75 × 70-mm-sized mass, infiltrating all the layers of jejunum wall, was found at the mesentery of the jejunum (Fig. 1c), and partial resection of the jejunum was then performed. Pathological examination revealed tumor cells with an acidophilic cells with an alveolar growth pattern (Fig. 2). Such observations were comparable with the primary gastric cancer’s pathological features. Immunohistochemical examination indicated that the tumor cells were positive for AFP and cytokeratin 18 and negative for cytokeratin 7 and 20. Consequently, we diagnosed the tumor to have metastasized from the AFP-GC resected 15 years previously. The preoperative serum AFP levels, which were postoperatively examined, were 17,447 ng/mL (normal range < 10 ng/mL). The patient was uneventfully discharged on the 15th postoperative day.

**Fig. 3:**
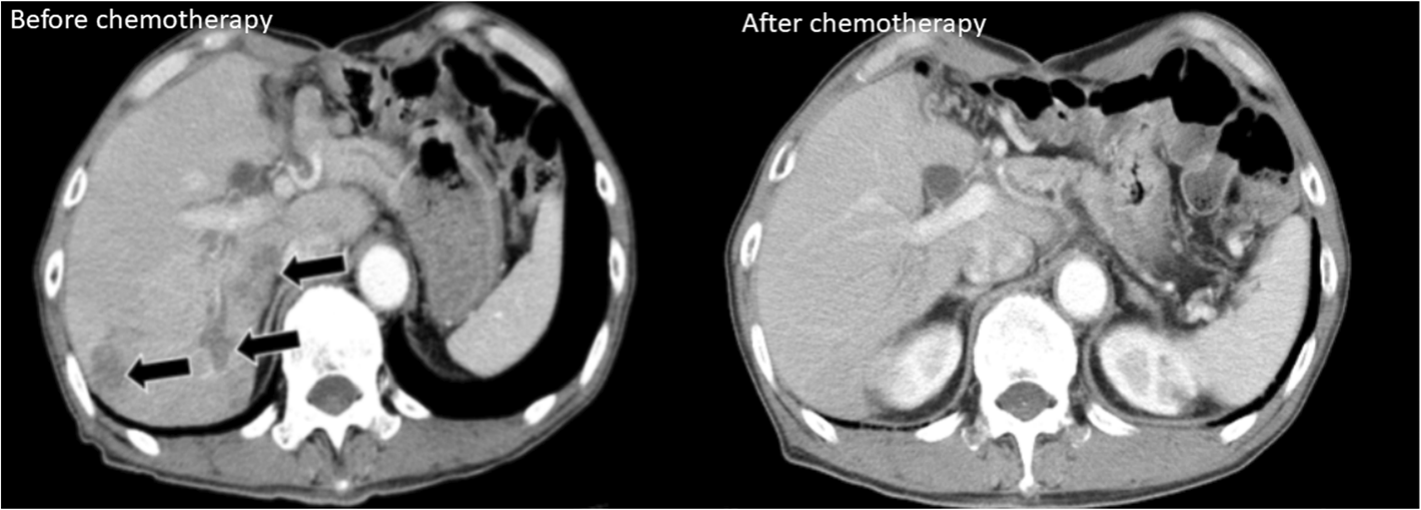
CT scan images before and after chemotherapy. Abdominal contrast-enhanced computed tomography revealed multiple low-density areas (arrows) 2 months after the second surgery. After 2 courses of chemotherapy, a CT scan revealed absence of all liver metastatic lesions

**Fig. 4:**
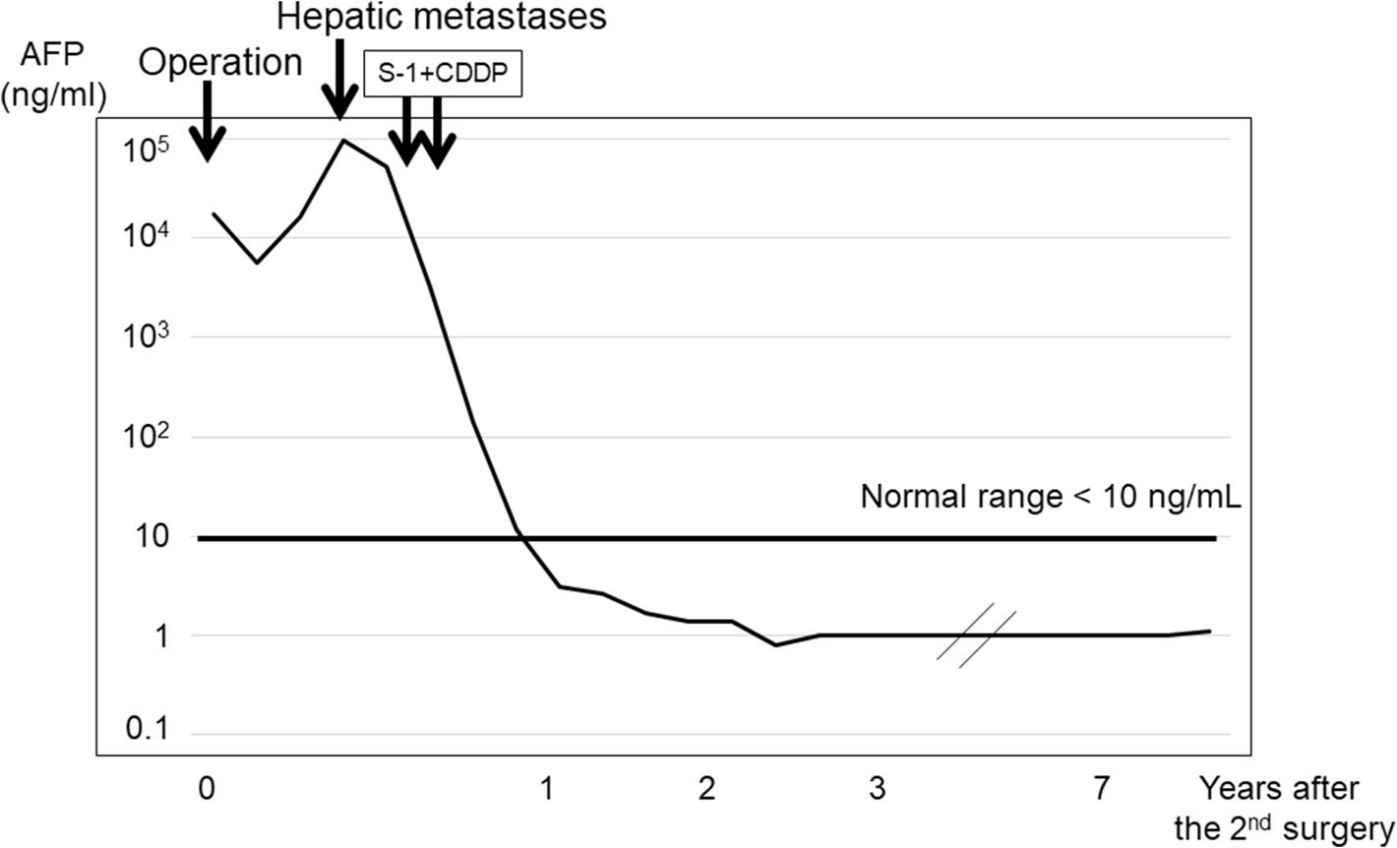
Clinical course and levels of serum alpha-fetoprotein. AFP, alpha-fetoprotein; CDDP cisplatin

Two months after the second surgery, CE-CT scan revealed multiple liver metastases (Fig. 3) and serum AFP level increased to 94,838 ng/mL (Fig. 4). Therefore, chemotherapy with S-1+CDDP was initiated. However, after two courses of therapy, the patient refused any further treatment owing to severe adverse effects (i.e., appetite loss and general fatigue). A CE-CT scan performed after two courses of chemotherapy revealed that all liver metastatic lesions disappeared. Additionally, serum AFP levels declined to normal range (1.0 ng/mL). Seven years after the second surgery, and without any chemotherapy, the patient is alive and well, without any recurrence.

## Discussion

Previous studies have reported a poor prognosis for AFP-GC with lymphatic and venous invasion, along with high rates of liver metastasis as compared with non-AFP-GC [4, 8, 9]. In a study by Liu et al., the authors reported that the 1-, 3-, and 5-year survival rates of AFP-GC were 53%, 35%, and 28%, respectively [6]. However, Kripp et al. described a long-term survival case of unresectable AFP-GC of the esophago-gastric junction with several liver metastases, which was successfully treated with capecitabine- and oxaliplatin-based combination therapy [10]. Of note, Shibata et al. also reported the case of a patient who underwent both distal gastrectomy for early AFP-GC and left hepatectomy 20 months later. The patient survived without any recurrence for over 11 years after the first diagnosis [11]. Thus, recently, there has been an increase in the number of reports on AFP-GC patients with long-term survival because of multimodal treatments [6, 12, 13].

Our case highlights three key learning points: first, the long-term (i.e., 15 years) dormancy following the initial gastrectomy until the identification of the solitary metastasis to the mesentery; second, the multiple liver metastases, which went unobserved for 15 years, were evident only 2 months after the second surgery; and last, the disappearance of liver metastases for 5 years following only two courses of chemotherapy with S-1+CDDP.

Gastric cancer usually recurs within the first 2 years of gastrectomy. Late recurrences are rare, with approximately < 1% cases recurring after 10 years [14]. Prognostic factors for early recurrence, such as clinical stage, tumor size, depth of invasion, and lymph node involvement, are not useful in predicting late cancer recurrence [15]. The phenomenon of late recurrence has been partially explained by tumor dormancy. Specifically, it has been shown that dormancy can be present in the form of an early stage in tumor development, as a micrometastasis, or as minimal residual disease post-surgical removal or treatment of the primary tumor [16–18]. In gastric cancer, minimal residual disease was revealed in the blood, bone marrow, negative lymph nodes, and peritoneal cavity [19]. Till date, transition mechanism from dormancy to recurrence has not been completely explained, particularly in gastric cancer. Although precise mechanisms remain to be elucidated, increasing evidence suggest that many cancer patients suffer from metastatic relapse several years after they have undergone radical surgery [18]. Thus, the second surgery reactivated the tumor cells which were dormant for 15 years. Additional studies aimed at better understanding of tumor dormancy are necessary to predict late recurrence and to strengthen the follow-up protocol for long-term survivors of gastric cancer.

In an earlier study, Adachi et al. analyzed 270 AFP-GC cases. The authors reported that the 5-year survival rate and median survival period in all patients were 22% and 14 months, respectively. However, they found that patients with curative gastrectomy comprised 42% of the study cohort and median survival period was 29 months [5]. Of note, Inoue et al. analyzed 53 AFP-GC cases reporting a 5-year survival rate of 34% (18/53) [20]. Our patient was alive 15 years after a curative distal gastrectomy, until the recurrence of mesentery of jejunum.

In Japan, SP therapy for unresectable and recurrent gastric cancers has been recognized as the standard therapy [21]. At the time of diagnosis, our patient was expected to have a poor prognosis due to recurrence of AFP-GC. However, only two courses of chemotherapy with SP were extremely effective on metachronous liver metastases. Surprisingly, the patient is alive without any sign of recurrence at 7 years after the second surgery. With the development of chemotherapy strategies, there are increasing cases who achieved complete response by only several courses of chemotherapy in unresectable gastric cancer [22, 23]. Further accumulation of cases is required for selecting such patients.

## Conclusion

Even if recurrence of AFP-GC is diagnosed, there are cases in which radical resection and chemotherapy are effective, as noted in the present case. Additional accumulation of AFP-GC cases is necessary to identify and select such cases.

## Data Availability

All data are available on request.

## Abbreviations

AFP-GC: Alpha-fetoprotein-producing gastric cancer
SP: Chemotherapy with S-1+CDDP
